# Infant and Toddler Feeding and Development: Habits for a Lifetime

**DOI:** 10.3390/nu18193113

**Published:** 2026-09-22

**Authors:** Maureen M. Black, Jigna M. Dharod

**Affiliations:** 1 Department of Pediatrics, University of Maryland School of Medicine, Baltimore, MD 21201, USA; 2 Department of Nutrition, School of Health and Human Sciences, University of North Carolina at Greensboro, Greensboro, NC 27412, USA; jmdharod@uncg.edu

## 1. Introduction

This Special Issue of *Nutrients* on Infant and Toddler Feeding and Development features feeding as a key element in young children’s early development. Feeding includes both what children eat (food or nutrients) and how children are fed (caregiver–child feeding practices). In addition to nutrition and feeding, sleep, play, and physical activity are typically part of children’s daily routines and contribute to early development [1]. Sleep refers to the amount of sleep that children need and receive. Play refers to children’s early explorations and interactions as they learn about their caregivers and their environment. Physical activity during infancy refers to children’s movements and motor competence as they develop both strength and motor skills. Thus, nutrition and feeding style, sleep, and play and physical activity comprise young children’s early lifestyle behaviors and contribute to their growth and development [1].

Children’s early growth and development are also influenced by biological theories. From a biological/gene perspective, including the Developmental Origins of Health and Disease (DOHaD), prenatal and postnatal factors such as nutrition and stress can influence the expression of genes guiding growth and health throughout life [2]. As illustrated by the Helsinki Birth Cohort Study, prenatal growth faltering followed by postnatal rapid weight gain has been associated with an increase in adult non-communicable diseases, specifically coronary artery disease and type 2 diabetes [3]. Early stunting (length-for-age z-scores < −2) is often caused by undernutrition and infections and has been associated with negative consequences in adult health, well-being, and economic productivity [4,5].

Children’s early lifestyle behaviors also contribute to adult health, well-being, and economic prosperity [1]. When lifestyle behaviors are healthy, they have been positively associated with adult physical and mental health and well-being [6] and have attenuated some of the negative consequences associated with early adversities [7]. The recognition that children’s lifestyle behaviors are modifiable and have long-term impacts has led to global investigations and research on feeding practices, sleep, and play and physical activity among young children, as illustrated by the papers in this Special Issue.

Early interventions have been shown to be particularly effective among young children who have experienced prenatal adversities, such as stunting, and children living in disadvantaged situations that may include barriers to healthy lifestyles [8,9]. Establishing sleep, play, and feeding routines in the early years, including during the first two years, has been an effective strategy to increase children’s likelihood of having healthy habits, including high-quality sleep and well-being, and low risk of overweight and obesity.

Habits are behaviors that are repeated frequently, often occur automatically, and require limited thinking [10]. They are often embedded in routines, initiated in response to a stimulus, such as breakfast, and associated with benefits, such as feelings of satiation and satisfaction [11]. Therefore, habits are often self-reinforcing and maintained, particularly when they are part of a consistent routine. Healthy habits for adults include regular exercise or physical activity, consistent high-quality sleep patterns, and a balanced diet including fruit, vegetables, and whole grains. These healthy habits are associated with normal weight and relatively low risk for chronic illnesses, including non-communicable diseases, such as diabetes type 2 and cardiovascular diseases [1]. In contrast, unhealthy habits for adults may include limited exercise or physical activity, irregular sleep patterns, and dietary choices that include overeating or eating excess sugar and fast foods. These unhealthy habits are associated with overweight or obesity, along with increased risk for diabetes, coronary artery disease and other chronic health problems.

Childhood overweight and obesity early in life (under 24 months of age) is associated with increased likelihood of unhealthy weight status in adulthood [12]. The consistency of weight status from childhood to adulthood has been at least partially explained by consistency and clustering in healthy and unhealthy lifestyle behaviors (e.g., fruits, vegetables, and outdoor play vs. discretionary consumption and television watching) from early childhood to adolescence [13], suggesting that interventions designed to promote healthy lifestyle behaviors during early childhood may help prevent non-communicable diseases in adulthood by improving the likelihood of continuing with healthy lifestyle behaviors later in life.

## 2. Strategies to Establish Healthy Habits in Early Childhood

Although providing care for young children can be joyous, caregiving may be demanding when infants have not established sleep patterns that enable caregivers to get adequate sleep themselves or when caregivers have multiple household and external responsibilities with limited support or resources. In such situations, routines may be particularly beneficial for young children and their caregivers. Many parenting programs are designed to help caregivers establish routines that incorporate healthy habits for their child and themselves [14].

### 2.1. Routines

Daily routines for young children’s basic activities, including eating, sleeping, and playing, can help children learn what to expect, enabling them to feel prepared and secure in a consistent routine [11]. As children learn to wait for an expected activity, their self-regulation improves by recognizing that the expected activities will occur even with delays. Routines also benefit caregivers by contributing to their overall organization, enabling them to plan activities for the day, and reducing potential power struggles with their child. When children are exposed to healthy choices, they become familiar with the healthy choices, especially if they can engage in the healthy choices with their caregivers.

### 2.2. Interactions

Children learn by observing caregivers’ daily behaviors and interacting with their caregivers in daily activities. Children imitate what they see and often enjoy eating and sharing food with caregivers. When caregivers describe their choices, children learn to solve problems in making decisions [11]. Young children crave their caregivers’ attention. By recognizing children’s signals and responding promptly and with nurturance, caregivers help young children establish the neural foundation needed for self-regulation and executive function. An emotionally supportive environment reduces stress and encourages children to explore and to build healthy relationships with others.

## 3. Conclusions

In summary, the articles in this Special Issue demonstrate the critical role that feeding plays in young children’s development, along with other lifestyle behaviors, including sleeping, play, and physical activity. Children who establish healthy lifestyle habits early in life are better prepared to practice healthy habits during childhood and into adulthood, increasing the likelihood for health, well-being, economic productivity, and avoiding chronic illnesses [15].

## Abbreviations

The following abbreviation is used in this Editorial:

DOHaD	Developmental Origins of Health and Disease
